# Circulating follistatin concentrations in adolescent PCOS: Divergent effects of randomized treatments

**DOI:** 10.3389/fendo.2023.1125569

**Published:** 2023-02-09

**Authors:** Marta Díaz, Francis de Zegher, Lourdes Ibáñez

**Affiliations:** ^1^ Endocrinology Department, Institut de Recerca Sant Joan de Déu, University of Barcelona, Barcelona, Spain; ^2^ Centro de Investigación Biomédica en Red de Diabetes y Enfermedades Metabólicas Asociadas, Instituto de Salud Carlos III, Madrid, Spain; ^3^ University of Leuven, Leuven, Belgium

**Keywords:** follistatin, PCOS, hepato-visceral fat, metformin, pioglitazone, spironolactone, flutamide

## Abstract

**Purpose:**

Follistatin is a glycoprotein that represses members of the transforming growth factor-β superfamily including activin. Higher follistatin levels have been associated with an increased risk for type 2 diabetes and with polycystic ovary syndrome (PCOS). In non-obese adolescent girls with PCOS, insulin sensitization results in a healthier endocrine-metabolic outcome than oral contraception (OC); we assessed whether those differences are underscored by changes in serum follistatin concentrations.

**Methods:**

Circulating follistatin, endocrine-metabolic markers and hepato-visceral fat were measured longitudinally in 72 girls with PCOS [age, 16 years; body mass index (BMI), 23 Kg/m^2^] randomized to receive PioFluMet [pioglitazone (7.5 mg/d), metformin (850 mg/d) and flutamide (62.5 mg/d), n=17]; EE-CA [an OC containing 35 µg ethinylestradiol (EE) and 2 mg cyproterone acetate (CA), n=17]; SPIOMET [Spironolactone (50 mg/d), pioglitazone (7.5 mg/d) and metformin (850 mg/d), n=18], or EE-LNG [an OC containing 20 µg EE and 100 mg levonorgestrel (LNG), n=20]. Twenty-eight age- and BMI-matched healthy girls served as controls.

**Results:**

Pre-treatment follistatin levels were similar in PCOS and controls. OCs raised serum follistatin after 6 months (6.8-fold vs 2.5-fold for EE-CA and EE-LNG, respectively). Neither SPIOMET nor PioFluMet changed follistatin levels. Follistatin correlated negatively with high-molecular weight adiponectin and positively with mean serum insulin concentrations during an oral glucose tolerance test at baseline, and with liver fat after 6 months.

**Conclusion:**

In girls with PCOS, follistatin levels rise significantly after 6 months on OCs and this increase associates to a worsening of markers of insulin resistance and to changes in liver fat.

## 1 Introduction

Adolescent polycystic ovary syndrome (PCOS) is a common endocrine disorder hallmarked by clinical and biochemical androgen excess and irregular menses. PCOS appears to be driven by ectopic lipid accumulation specially in the liver that essentially originates from a mismatch between (reduced) prenatal adipogenesis and (augmented) postnatal lipogenesis, resulting in central obesity, insulin resistance, non-alcoholic fatty liver disease (NAFLD), and low-grade inflammation (1). There is no approved therapy for PCOS, but girls are commonly treated with oral contraceptives (OCs), even if not at pregnancy risk. OCs revert the signs and symptoms of androgen excess but fail to address the core problem, and upon treatment discontinuation, there is a rebound of hyperandrogenism and oligo‐anovulation. An alternative approach under investigation are the low-dose combinations of insulin sensitizers and anti-androgens with additive effects that switch ectopic fat to eutopic depots, thereby normalizing ovarian function and potentially reducing the risk of long-term co-morbidities. We performed three pilot studies in non-obese girls with PCOS comparing the effects of such combinations with those of OCs. In the first clinical trial (ISRCTN45546616), girls were randomized to receive for one year a low-dose combination of two insulin sensitizers [pioglitazone (7.5 mg/d), and metformin (850 mg/d)] and one anti-androgen [flutamide (62.5 mg/d)] (PioFluMet), or an OC containing ethinylestradiol- cyproterone acetate [EE-CA; 35 µg of EE plus 2 mg of CA for 21/28 d, placebo for 7/28 d; Diane 35 Diario^®^, Bayer-Schering, Madrid, Spain]. Both treatments decreased similarly androgen excess, but PioFluMet had more benefits on cardiometabolic parameters and adipose tissue expression of genes related to inflammation, fat accretion and lipoprotein metabolism (2, 3). In the other two studies (ISRCTN29234515 and ISRCTN11062950), girls were randomized to receive for one year a low-dose combination of spironolactone (50 mg/d), pioglitazone (7.5 mg/d), and metformin (850 mg/d) (SPIOMET) or an OC containing EE-levonorgestrel [EE-LNG; 20 µg of EE plus 100 mg of LNG for 21/28 d, placebo for 7/28 d; Loette Diario^®^, Pfizer, Madrid, Spain] (4, 5). The pooled results of these two studies disclosed that SPIOMET intervention is followed by a healthier metabolic status [less insulin resistance and C-reactive protein (CRP) concentrations, less hepato-visceral fat, and higher high-molecular-weight (HMW) adiponectin], and by more ovulations than treatment with OCs (5).

Follistatin was initially identified in and isolated from follicular fluid based on its inhibition of pituitary FSH secretion (6). Later on, follistatin was characterized as a reproductive hormone inhibiting the secretion of members of the transforming growth factor (TGF)-β family of proteins, including activin and inhibin, and as an enhancer of muscle mass through the inhibition of myostatin (7). In humans, follistatin derives mainly from the liver and augmented follistatin levels have been associated with an increased risk for type 2 diabetes, independently of established risk markers (8). Recently, follistatin has been considered to play a role in the etiology of PCOS, as women diagnosed with this disorder depict increased follistatin concentrations *versus* controls, independently of body mass index (BMI) (9, 10). However, those studies were conducted in adult women with a wide age-range diagnosed with PCOS using heterogeneous criteria. Here, we assessed whether the divergent metabolic effects of OCs and low-dose PioFluMet or SPIOMET in adolescent girls with PCOS over the first 6 months of treatment associate to changes in circulating follistatin levels.

## 2 Materials and methods

### 2.1 Study design

The study population consisted of 72 non-obese adolescent girls with PCOS belonging to the above described clinical trials [n=17 and n=17 receiving PioFluMet and EE-CA respectively; n=18 and n=20 receving SPIOMET and EE-LNG respectively); mean age, 16 years; mean BMI, 23 kg/m^2^; all of them were at last 2 years beyond menarche. The girls were recruited in the Adolescent Endocrinology Unit of Sant Joan de Déu University Hospital, Barcelona, Spain. Randomization was performed with the SealedEnvelop program (Sealed Envelop Ltd., London, UK) (http://www.SealedEnvelop.com), using random permuted blocks with strata for age and BMI (10).The inclusion criteria were (2–5): 1) hirsutism (score > 8 on Ferriman-Gallwey scale); 2) amenorrhea (no menses for more than 3 months) or oligomenorrhea (menstrual intervals >45 days); 3) absence of sexual activity throughout the study duration (and thus, no need for contraception). Exclusion criteria were: 21‐hydroxylase deficiency; glucose intolerance or diabetes; evidence of thyroid, liver, or kidney dysfunction; hyperprolactinemia; and prior use of medications affecting gonadal/adrenal function, or carbohydrate/lipid metabolism.

Twenty-eight age- and BMI-matched healthy girls recruited from nearby schools served as controls. All had regular cycles, were non-hirsute, and had normal serum glucose, lipids and androgens.

The PioFluMet study (ISRCTN45546616) and both SPIOMET studies (ISRCTN29234515 and ISRCTN11062950) were conducted after approval by the Institutional Review Board of Sant Joan de Déu University Hospital, after written consent by parents, and assent by each of the study girls, including the healthy controls who allowed to derive indicative values.

### 2.2 Assessments

Height and weight were measured and BMI calculated. Blood samples were obtained-in the follicular phase or after 2 months of amenorrhea- in the morning after an overnight fast. Serum glucose was measured by the glucose oxidase method. Insulin, testosterone and sex hormone binding globulin (SHBG) were assayed by immunochemiluminiscence (DPC IMMULITE 2500, Siemens, Erlangen, Germany); intra- and inter-assay coefficients of variation (CVs) were <10%; HDL-cholesterol, LDL-cholesterol and triglycerides were assessed by an enzymatic method and C-reactive protein (CRP) was measured with a hightly sensitive method (Architect c8000 autoanalyzer, Abbott laboratories, North Chicago, IL). Homeostasis model assessment for insulin resistance (HOMA-IR) was calculated as fasting insulin (mU/L) x fasting glucose (mmol/L)/22.5.

Circulating follistatin and HMW-adiponectin were measured by specific ELISAs (R&D Systems, Minneapolis, USA) with intra- and inter-assay CVs <9% for both assays.

Abdominal fat partitioning (subcutaneous and visceral fat areas) as well as liver fat were assessed by magnetic resonance imaging (MRI) using a multiple-slice MRI 1.5 Tesla scan (Signa LX Echo Speed Plus Excite, General Electric, Milwaukee, WI), as reported (2–4).

### 2.3 Statistics

Statistical analysis were performed with GraphPad Prism 6.01. Results are expressed as mean ± SEM. Variables were checked for normality using the Kolmogorov–Smirnov test prior to analyses. Comparisons within groups were performed using paired t-test. For between groups differences, unpaired t-test or Man-Whitney U test were used for normally distributed or nonparametric variables, respectively.

Correlation analysis was used to study the associations between follistatin concentrations and auxological, endocrine-metabolic, and body composition parameters. Three outliers were identified by the interquartile range method; correlations are presented for n=69 out of 72 girls. The level of significance was set at p< 0.05.

## 3 Results

Both PioFluMet and SPIOMET reduced androgen excess within 6 months towards normal, similarly to EE-CA or EE-LNG. However, only treatment with PioFluMet or SPIOMET -but not with OCs- reduced both the hepatic fat excess and insulin resistance (**Table 1**).

**Table 1:** Data from adolescent girls with androgen excess who were randomized to receive ethinylestradiol-cyproteroneacetate [EE-CA (N=17)], low-dose pioglitazone-flutamide-metformin [PioFluMet (N=17)], ethinylestradiol-levonorgestrel [EE-LNG(N=20)] or low-dose spironolactone-pioglitazone-metformin [SPIOMET (N=18)] for 6 months, and from age and BMI-matched-healthy control girls (N=28).

			Ethinylestradiol-Cyproteroneacetate [EE-CA (N=17)]			Pioglitazone-Flutamide-Metformin [PioFluMet (N=17)]			Ethinylestradiol-Levonorgestrel [EE-LNG (N=20)]			Spironolactone-Pioglitazone-Metformin [SPIOMET (N=18)]		
	Controls (N=28)	PCOS (N=73)	Baseline	6 mo	Δ 0-6 mo	Baseline	6 mo	Δ 0-6 mo	Baseline	6 mo	Δ 0-6 mo	Baseline	6 mo	Δ 0-6 mo
Age (yr)	16.3 ± 0.3	15.8 ± 0.2	15.9 ± 0.3	–	–	16.5 ± 0.3	–	–	15.7 ± 0.3	–	–	15.4 ± 0.3	–	–
BMI (kg/m^2^)	22.6 ± 0.4	23.5 ± 0.4	23.1 ± 0.6	23.6 ± 0.7^a^	0.5 ± 0.2	23.2 ± 0.5	23.3 ± 0.5	0.1 ± 0.2	24.6 ± 0.9	25.0 ± 0.9	0.4 ± 0.3	23.1 ± 0.7	23.1 ± 0.7	0.0 ± 0.2
SHBG (nmol/L)	67 ± 9	29 ± 2^***^	23 ± 3	159 ± 10^c^	136 ± 9	28 ± 3	35 ± 4^a^	7 ± 3	32 ± 3	60 ± 5^c^	28 ± 5	30 ± 3	30 ± 2	0 ± 2^f^
Testosterone (ng/dL)	29 ± 2	55 ± 3^***^	57 ± 7	32 ± 4^c^	-25 ± 5	62 ± 7	43 ± 5^b^	-19 ± 6	54 ± 5	29 ± 3^c^	-25 ± 5	47 ± 3	31 ± 3^c^	-16 ± 4
FAI	1.5 ± 0.3	8.3 ± 0.6^***^	10.8 ± 1.8	1.1 ± 0.4^c^	-9.7 ± 1.5	9.5 ± 1.4	4.9 ± 0.7^c^	-4.6 ± 0.9^e^	6.7 ± 0.8	1.9 ± 0.2^c^	-4.8 ± 0.8	6.5 ± 1.0	4.3 ± 0.5^b^	-2.2 ± 0.7^d^
Fasting insulin (µU/mL)	4.9 ± 0.7	11.0 ± 0.8^***^	9.7 ± 1.1	8.1 ± 1.3	-1.6 ± 1.7	10.2 ± 1.9	5.5 ± 1.3^a^	-4.7 ± 1.8	13.5 ± 1.8	15.0 ± 1.5	1.5 ± 1.1	10.1 ± 1.5	8.9 ± 1.1	-1.2 ± 1.0
HOMA-IR	1.1 ± 0.2	2.3 ± 0.2^**^	2.0 ± 0.3	1.7 ± 0.3	-0.3 ± 0.4	2.1 ± 0.4	1.2 ± 0.3^a^	-0.9 ± 0.4	2.9 ± 0.4	3.3 ± 0.4^a^	0.4 ± 0.3	2.1 ± 0.3	1.8 ± 0.2	-0.3 ± 0.2^d^
LDL-cholesterol (mg/dL)	83 ± 4	84 ± 2	81 ± 3	102 ± 5^c^	21 ± 5	80 ± 5	82 ± 3	2 ± 3^e^	90 ± 3	105 ± 5^c^	15 ± 4	84 ± 6	84 ± 5	0 ± 3^e^
HDL-cholesterol (mg/dL)	53 ± 2	52 ± 1	50 ± 2	61 ± 3^c^	11 ± 2	56 ± 3	57 ± 2	1 ± 2^f^	51 ± 2	52 ± 3	1 ± 2	51 ± 2	54 ± 2^a^	3 ± 1
Triglycerides (mg/dL)	53 ± 3	67 ± 6	89 ± 20	130 ± 20^b^	41 ± 21	65 ± 11	67 ± 6	1 ± 7	58 ± 5	65 ± 6	7 ± 5	59 ± 5	58 ± 4	-1 ± 5
C-Reactive Protein (mg/L)	0.7 ± 0.2	1.2 ± 0.1^*^	0.9 ± 0.2	1.7 ± 0.3^a^	0.8 ± 0.3	1.0 ± 0.2	0.4 ± 0.1^b^	-0.6 ± 0.2^f^	1.4 ± 0.3	2.4 ± 0.5^a^	1.0 ± 0.4	1.4 ± 0.4	0.5 ± 0.1^a^	-0.9 ± 0.4^e^
HMW adiponectin (mg/L)	10 ± 1	9 ± 1	16 ± 3	11 ± 1^a^	-5 ± 2	11 ± 2	18 ± 1^c^	7 ± 2^f^	6 ± 1	9 ± 2	3 ± 2	6 ± 1	15 ± 2^a^	9 ± 2
Follistatin (ng/mL)	0.7 ± 0.1	0.7 ± 0.1	0.6 ± 0.2	3.9 ± 0.8^c^	3.3 ± 0.8	0.4 ± 0.1	0.5 ± 0.1	0.1 ± 0.1^f^	0.8 ± 0.1	2.0 ± 0.4^b^	1.2 ± 0.4	1.1 ± 0.3	1.0 ± 0.1	-0.1 ± 0.3^e^
MRI subcutaneous Fat (cm^2^)	98 ± 22	152 ± 10	130 ± 16	145 ± 18^a^	15 ± 7	127 ± 13	123 ± 14	-4 ± 6^d^	193 ± 26	195 ± 25	2 ± 8	152 ± 17	147 ± 17	-5 ± 6
Visceral Fat (cm^2^)	29 ± 3	37 ± 2	32 ± 2	35 ± 2	3 ± 2	36 ± 3	31 ± 2^b^	-5 ± 2^e^	40 ± 4	41 ± 4	1 ± 2	39 ± 3	35 ± 2	-4 ± 3
Liver fat (%)	11.5 ± 2.8	16.4 ± 0.7	17.0 ± 1.6	18.9 ± 1.5	1.9 ± 1.5	15.1 ± 1.5	10.1 ± 1.2^b^	-5.0 ± 1.7^e^	16.3 ± 1.5	20.1 ± 1.6^a^	3.8 ± 1.5	17.2 ± 1.3	11.0 ± 1.1^c^	-6.2 ± 1.0^f^

Values are mean ± SEM. MRI, magnetic resonance imaging; BMI, bone mineral density; SHBG, sex hormone-binding globulin; FAI, free androgen index; HOMA-IR, homeostasis model assessment insulin resistance; HMW, high-molecular weight.

^*^p <0.05, ^**^p<0.01 and ^***^p≤0.0001 between PCOS at start and healthy control girls.

^a^p <0.05, ^b^ p≤0.01 and ^c^p ≤0.001 within subgroups for 0-6 mo change (Δ).

^d^p <0.05, ^e^ p ≤0.01, ^f^ p ≤0.001 between EE-CA and PioFluMet subgroup & EE-LNG and SPIOMET subgroup for 0-6 mo changes (Δ).

Pre-treatment follistatin levels were similar in girls with PCOS and in controls (0.7 ± 0.1 ng/mL in both subgroups). OCs significantly raised serum follistatin after 6 months; this increase was higher with EE-CA than with EE-LNG (6.8-fold vs 2.5-fold *versus* baseline; p<0.0006 and p<0.003, respectively). Neither SPIOMET nor PioFluMet had effects on follistatin levels (**Table 1**).

**Figure 1:**
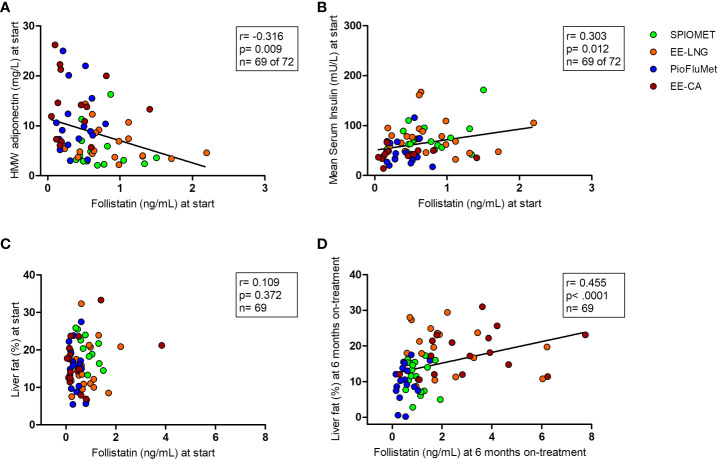
Bivariate correlations between circulating follistatin and, respectively, HMW-adiponectin and mean insulin concentration during an oral glucose tolerance test at baseline [panels **(A)** and **(B)**], and liver fat at baseline and after 6 months on treatment [panels **(C)** and **(D)**], in adolescent girls with PCOS (N=69), randomized to receive PioFluMet, SPIOMET, EE-CA or EE-LNG.

At baseline, circulating follistatin correlated negatively with HMW-adiponectin (r=-0.316, p=0.009) and positively with mean insulin levels during an oral glucose tolerance test (OGTT; r= 0.303, p= 0.012) [**Figures 1A, B**]. Pre-treatment liver fat was not related to follistatin levels; however, after 6 months on treatment, liver fat directly and significantly associated with serum follistatin [**Figures 1C, D**)].

## 4 Discussion

To our knowledge this is the first longitudinal study comparing the effects of OCs *vs* insulin sensitization on circulating follistatin levels in non-obese adolescent girls with PCOS. Our results disclose that follistatin concentrations increase with OC therapy but remain unchanged with PioFluMet or SPIOMET combinations.

Girls with PCOS and without obesity showed similar serum follistatin concentrations as compared to age- and BMI-matched healthy girls, in contrast to previously reported data (9, 10). However, those dissimilar data should be interpreted with caution, because in the meta-analysis performed pooling the results of eight studies comparing follistatin levels in women with PCOS and in healthy controls with a wide range of age and BMI, the association between follistatin and PCOS could vary when the average age difference between PCOS patients and controls was very wide (10). Also, in patients with nonalcoholic simple steatosis – which is common in PCOS- follistatin levels were found to be comparable to those of healthy controls regardless of BMI (11).

Overall, our data agree with previous studies showing a raise in follistatin concentrations after treatment with OCs containing different combinations of estroprogestagens (12, 13). Here, we report for the first time that the increase in follistatin levels is less pronounced when the OC contains LNG as progestagen instead of CA, and that low-dose combinations of insulin sensitizers and anti-androgens such as PioFluMet and SPIOMET that improve metabolic health in adolescent PCOS, have no effects on circulating follistatin. To our knowledge, there are no other studies directly comparing the effects of different OC combinations or the effects of new progestagens like dienogest on follistatin concentrations. Although the increase in follistatin levels may be attributted to increased hepatocyte secretion, those studies might unveil the existence of additional pathways through which estro-progestagens can induce elevations of follistatin.

In our population of girls with PCOS, follistatin associated positively with mean serum insulin concentrations during an OGTT and with liver fat. These findings agree with a previous report showing that the rise in follistatin levels is capable of inducing adipose tissue insulin resistance and thus could increase the risk for type 2 diabetes (8). On the other hand, the inverse association found between circulating follistatin and HMW-adiponectin - an adipokine with insulin-sensitizing and cardio-protective properties (5), is in alignment with the inhibitory effect of HMW-adiponectin on hepatic follistatin secretion (14).

Limitations of the present study include the relative small number of patients allocated to each intervention and the lack of follistatin results during the period off intervention. The strengths include the longitudinal design, the homogenity of the study population and the assessment of two different interventions with insulin sensitizing therapies (PioFluMet and SPIOMET), and with OCs as well (EE-CA and EE-LNG); i.e., with two approaches with divergent effects on metabolic health.

In conclusion, in girls with PCOS, follistatin levels rise significantly after 6 months on OCs and this increase associates to a worsening of markers of insulin resistance and to the changes in ectopic fat depots, specifically in liver fat.

## Data availability statement

The raw data supporting the conclusions of this article will be made available by the authors, without undue reservation.

## Ethics statement

The studies involving human participants were reviewed and approved by Ethics Committee Pediatric Research Institute Sant Joan de Déu. Written informed consent to participate in this study was provided by the participants’ legal guardian/next of kin.

## Author contributions

MD contributed to literature search, design of Figures and Tables, data collection, data analysis and interpretation and wrote the manuscript. LI contributed to study design, literature search, data analysis and interpretation and reviewed/edited the manuscript. FZ contributed to data interpretation and reviewed/edited the manuscript. All authors contributed to the article and approved the submitted version.
